# Modulation of viral replication in macrophages persistently infected with the DA strain of Theiler's murine encephalomyelitis virus

**DOI:** 10.1186/1743-422X-5-89

**Published:** 2008-08-04

**Authors:** Stephane Steurbaut, Ellen Merckx, Bart Rombaut, Raf Vrijsen

**Affiliations:** 1 Department of Pharmaceutical Biotechnology and Molecular Biology, Vrije Universiteit Brussel, Brussels, Belgium

## Abstract

**Background:**

Demyelinating strains of Theiler's murine encephalomyelitis virus (TMEV) such as the DA strain are the causative agents of a persistent infection that induce a multiple sclerosis-like disease in the central nervous system of susceptible mice. Viral persistence, mainly associated with macrophages, is considered to be an important disease determinant that leads to chronic inflammation, demyelination and autoimmunity. In a previous study, we described the establishment of a persistent DA infection in RAW macrophages, which were therefore named DRAW.

**Results:**

In the present study we explored the potential of diverse compounds to modulate viral persistence in these DRAW cells. Hemin was found to increase viral yields and to induce cell lysis. Enviroxime and neutralizing anti-TMEV monoclonal antibody were shown to decrease viral yields, whereas interferon-α and interferon-γ completely cleared the persistent infection. We also compared the cytokine pattern secreted by uninfected RAW, DRAW and interferon-cured DRAW macrophages using a cytokine protein array. The chemokine RANTES was markedly upregulated in DRAW cells and restored to a normal expression level after abrogation of the persistent infection with interferon-α or interferon-γ. On the other hand, the chemokine MCP-1 was upregulated in the interferon-cured DRAW cells.

**Conclusion:**

We have identified several compounds that modulate viral replication in an *in vitro *model system for TMEV persistence. These compounds now await further testing in an *in vivo *setting to address fundamental questions regarding persistent viral infection and immunopathogenesis.

## Background

The DA strain of Theiler's murine encephalomyelitis virus (TMEV), a picornavirus belonging to the *Cardiovirus *genus, is the causative agent of a biphasic disease in the central nervous system (CNS) of susceptible mice. In a first phase, the virus infects neurons and causes an acute but mild encephalomyelitis that lasts for one to two weeks. This is followed by a second phase, during which the virus infects glial cells of the spinal cord's white matter and that is characterized by chronic inflammation and demyelination resembling the human disease multiple sclerosis (MS) [1-3]. The virus persists lifelong in infected mice, with macrophages representing the main viral reservoir [4,5]. Although various immune responses are activated to resist the viral infection, these defense mechanisms are also suspected to inflict myelin damage, e.g. anti-TMEV antibodies could cross-react with myelin components such as galactocerebroside, resulting in virus-induced autoimmune myelin destruction [6,7]. Infected mice also mount a virus-specific CD4^+ ^Th1 lymphocyte response that contributes to demyelination via bystander damage induced by a delayed-type hypersensitivity response [8]. Later, myelin epitopes, released as a consequence of tissue destruction, lead to the activation of myelin-specific Th1 cells that trigger autoimmunity [9]. Apart from CD4^+ ^Th1 lymphocytes, CD8^+ ^T cells have also been implicated in autoimmunity. Borrow *et al *[10] demonstrated that CD8^+ ^T cells are important for viral clearance, but these cells may also be critical effectors that aggravate the demyelination [11-13]. In addition, TMEV infection triggers the production of multiple cytokines and chemokines that likely initiate, enhance and/or perpetuate the inflammatory responses leading to demyelination [14-17]. Because demyelination is associated with ongoing CNS infection, viral persistence is assumed to be necessary for this pathology to develop. In addition, some mouse strains develop encephalomyelitis after DA infection, but are resistant to demyelination due to elimination of the virus [18]. However, once autoimmunity is established in susceptible mice, it remains unknown whether it can be self-perpetuating when the virus would be cleared, a question so far unaddressed due to the lack of *Cardiovirus *inhibitors [19].

Previously, we have shown that Theiler's DA strain readily establishes a long-term persistent infection in RAW264.7 macrophages (RAW). This persistently infected continuous cell line has been termed DRAW. The infection was productive and showed only restricted cytopathic effects [20]. The purpose of the present study was to evaluate different treatments for their potential to modulate viral persistence in DRAW cells, whereby both the downregulation as well as the upregulation of the infection were considered. In addition, we examined the macrophages' cytokine and chemokine expression pattern, before and after recovery from persistent infection.

## Results

### Screening of compounds for a modulating effect on viral persistence in DRAW cells

In a previous study, we reported DRAW macrophage cell cultures to be persistently infected with the DA strain [20]. Here, we explored the possibility to modulate viral persistence in DRAW cells using various compounds. These compounds were selected in function of an anticipated or established effect on picornavirus replication (Table 1). DRAW cells, cultivated for approximately 3 months (20 passages) and seeded at 2.5 × 10^4 ^cells/well in 96-well plates, were subjected to different concentrations (mostly 5-fold dilutions) of the various compounds. To make a distinction between compound-induced cytotoxic effects and virus-induced cytopathic effects, untreated DRAW and compound-treated RAW as well as untreated RAW cells were used as controls in the microscopic evaluation of cytotoxicity. After 48 and 96 h, DRAW culture supernatants and cells were collected, and following three freeze-thaw cycles, submitted to plaque assay. Only samples without microscopic evidence of compound-induced cytotoxicity were titrated. On the basis of the plaque assay results, the compounds were categorized into three classes: (1) 2-aminopurine nitrate (2-AP), 2-furylmercury chloride (2-FMC), 5-(3,4-dichlorophenyl) methylhydantoin (hydantoin), levamisole, *N*^G^-nitro-L-arginine methyl ester (L-NAME), *N*^G^-monomethyl-L-arginine (L-NMMA) and pirodavir had no influence on virus yields compared to those from untreated DRAW cells, (2) hemin led to an increase of virus titers, and (3) neutralizing anti-TMEV VP1 monoclonal antibody (mAb), enviroxime (ENV), recombinant murine interferon (IFN)-α and recombinant murine IFN-γ led to a decrease of infectivity (Table 1). Of the compounds that affected virus titers in DRAW cells, we determined the concentration that increased or decreased virus yield by fifty percent and we named this the effective concentration_50 _(EC_50_) (Table 1). We also established the effective dose (ED) of these compounds, i.e., the concentration that maximally affected virus yield from DRAW cells (Table 1).

**Table 1:** Listing of the compounds screened for a modulating effect on viral persistence in DRAW cells.

Compound	Established effect, virus (strain)	Reference	Effect on TMEV yield from DRAW	C_max_^d^	
2-AP	↑^a^, TMEV (GDVII)	[21]	-^c^	200 μg/ml	
L-NAME	↑, CVB (CVB3)	[22]	-	250 μg/ml	
L-NMMA	↑, CVB (CVB3)	[23]	-	250 μg/ml	
2-FMC	↓^b^, HRV (HRV2)	[24]	-	0.1 μg/ml	
hydantoin	↓, PV (Mahoney)	[25]	-	20 μg/ml	
levamisole	↓, EMCV	[26]	-	200 μg/ml	
pirodavir	↓, HRV (HRV9)	[27]	-	10 μg/ml	

Compound	Established effect, virus (strain)	Reference	Log_10 _maximal increase or decrease of TMEV yield from DRAW^e^	EC_50 _^e^	ED^f^

hemin	↑, PV (Mahoney)	[28]	↑; 0.99 ± 0.23	13 μg/ml	65 μg/ml
anti-TMEV mAb	↓, TMEV (DA, GDVII)	[29]	↓; 1.12 ± 0.04	1:250 dilution	1:10 dilution
enviroxime	↓, HRV (HRV31)	[30]	↓; 0.99 ± 0.13	0.1 μg/ml	0.316 μg/ml
IFN-α	↓, TMEV (DA)	[21]	↓; 4.89 ± 0.23	10 ng/ml	250 ng/ml
IFN-γ	↓, TMEV (DA)	[21]	↓; 5.89 ± 0.49	0.2 ng/ml	25 ng/ml

^a ^↑: increase of infectivity; ^b ^↓: decrease of infectivity; ^c ^-: no influence on infectivity; ^d ^C_max_: highest, non-cytotoxic concentration; ^e ^EC_50_: effective concentration_50 _= concentration of the compound inducing a twofold increase or decrease of infectious virus yield from DRAW cells, as determined by titration in L929 cells; ^f ^ED: effective dose = concentration that maximally affected infectious virus yield from DRAW cells; CVB: coxsackievirus B; EMCV: encephalomyocarditis virus; HRV: human rhinovirus; PV: poliovirus; TMEV: Theiler's murine encephalomyelitis virus. Data are the mean result of duplicate samples from two independent experiments ± standard deviation

Subsequently, a more detailed study with the compounds that induced a modulating effect on virus replication in DRAW cells was performed. A similar modus operandi was followed for each of these compounds. DRAW cells, cultivated in 96-well plates at 2.5 × 10^4 ^cells/well, were treated with the ED of each compound. Culture supernatants and cells were harvested at the start of the experiments and each following 24 h during 4 days, where after the infectivity was determined by plaque assay. In parallel, and in addition to the microscopic evaluation, each compound's ED was further tested in detail for cytotoxicity using the CellTiter-Blue cell viability assay that measures cellular metabolic activity. Compound-treated RAW as well as untreated DRAW and untreated RAW macrophages were again used as reference. Cytotoxicity was also assessed each 24 h during 4 days. As the EDs revealed to be non-toxic (results not shown), the results of the viability assay are only discussed where relevant. In the following paragraphs, a more detailed analysis of the results obtained with the different compounds is presented.

### Hemin upregulates virus replication and induces lysis of DRAW cells

**Figure 1:**
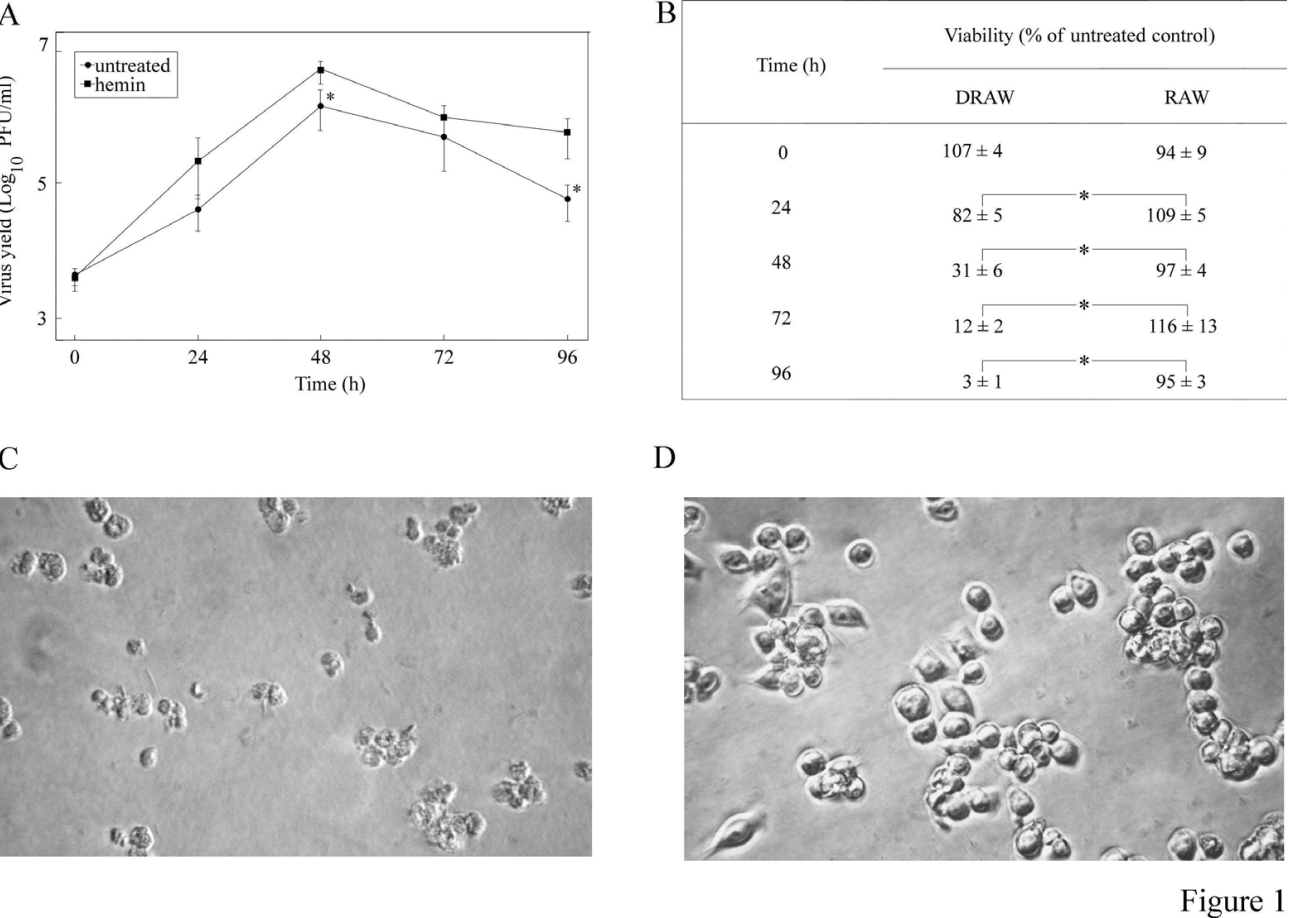
**Effect of hemin on DRAW and RAW cells**. Cells, seeded at a density of 2.5 × 10^4 ^cells/well in 96-well plates, were treated with 65 μg/ml hemin or untreated. (A) Virus yield from DRAW cells was measured in function of time by plaque assay in L929 cells. Data are the mean result of duplicate samples from two independent experiments ± standard deviation; * *P *< 0.05 (unpaired Student's *t *test between untreated and compound-treated samples). (B) Cell viability of hemin-treated cells was assayed using Promega's CellTiter-Blue kit on triplicate samples and expressed as a percentage of the values from untreated control cells ± standard deviation; * *P *< 0.05 (unpaired Student's *t *test between hemin-treated RAW and DRAW cells. Phase-contrast images of (C) hemin-induced lysis of DRAW cells and (D) normal appearing hemin-treated RAW cells. Magnification: 400×.

Benton *et al *[28] have shown that hemin enhances poliovirus replication in persistently infected K562-Mu erythroleukemia cells supposedly resulting from an increase in host shut-off due to protease-induced cleavage of the translation initiation factors eIF-4G and eIF-2α. In addition, hemin has been implicated in downregulation of the IFN signaling pathway [31-33]. These observations prompted us to investigate the effect of hemin on DRAW cells. Treatment of DRAW macrophages with 65 μg/ml hemin resulted in an average fivefold increase of virus titers that was maintained until the end of the experiment (Figure 1A). Moreover, hemin treatment led to a gradual decrease of the DRAW's viability over time (Figure 1B), resulting in the lysis of nearly all cells after 4 days (Figure 1C). In contrast, hemin-treated RAW (Figure 1B and 1D) and untreated DRAW cells (results not shown) remained fit, proving that the effect of hemin on DRAW cells was not due to compound-induced cytotoxicity, but probably resulted from the upregulation of viral replication and/or spread of the infection with a concomitant increase of virus-induced cytopathic effects.

### Enviroxime and anti-TMEV mAb decrease virus replication in DRAW cells

**Figure 2:**
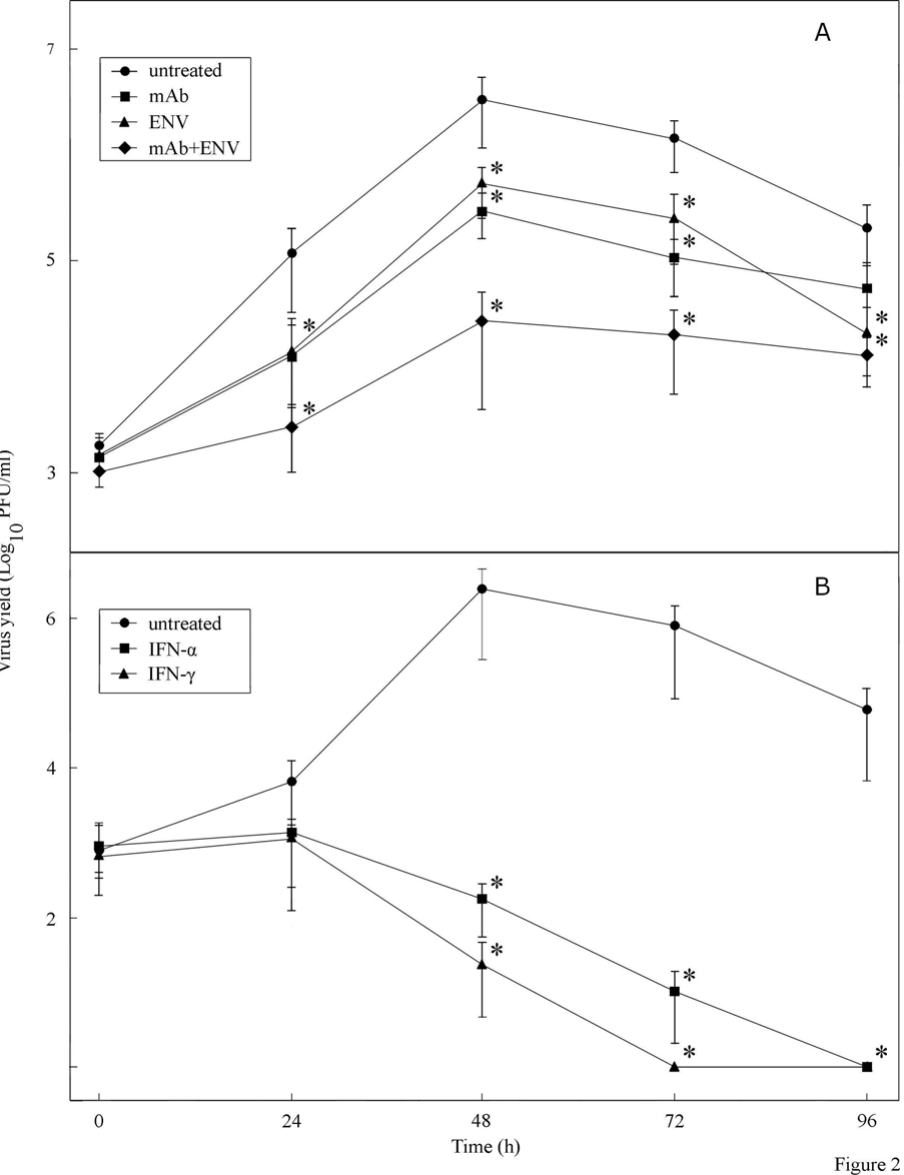
**Antiviral effect of compounds in DRAW cells in function of time**. (A) mAb (1:10 dilution), ENV (0.316 μg/ml) and mAb + ENV; (B) IFN-α (250 ng/ml) and IFN-γ (25 ng/ml). Controls consist of untreated cells. Cells were cultivated in 96-well plates at a density of 2.5 × 10^4 ^cells/well and virus yield was measured by plaque assay. Data are the mean result of duplicate samples from two independent experiments ± standard deviation; * *P *< 0.05 (unpaired Student's *t *test between untreated and compound-treated samples).

Enviroxime has been shown to exert an antipicornaviral effect on poliovirus and rhinoviruses through inhibition of viral RNA synthesis [34]. Neutralizing mAbs bind to virions thereby interfering with processes such as attachment, entry or uncoating, which results in a decrease of the infectivity [35]. We examined these compounds for their potential to reduce virus titers in DRAW macrophages by treating the cells either with enviroxime (0.316 μg/ml; the highest non-cytotoxic dose) or with a neutralizing anti-TMEV mAb recognizing VP1 (1:10 dilution), as well as with the combination of both. Compared to untreated DRAW cells, treatment with enviroxime or with anti-TMEV mAb, both led to an average decrease of the infectivity with nearly 1 log_10 _during the 4 days period that the experiment was carried out (Figure 2A). The combination of enviroxime with anti-TMEV mAb resulted in a decrease of the virus yield by about 2 log_10_. Although the addition of enviroxime and anti-TMEV mAb alone, or their combination decreased viral replication in DRAW cells, none of these treatments was able to cure the macrophage cell cultures from their persistent infection.

### IFN-α and IFN-γ clear DRAW cells of persistent viral infection

IFNs are key mediators of the innate antiviral immune response that are produced upon viral infection. They exert their antiviral effects through the induction of proteins such as the 2',5'-oligoadenylate synthetase, the double-stranded RNA-dependent protein kinase and the Mx proteins that mediate antiviral activity (for a review, see [36]).

Previously, we have reported that IFN-α and IFN-γ contribute to the antiviral response of RAW macrophages against TMEV, whereas this could not be demonstrated for IFN-β [21].

Here, we investigated the antiviral effect of IFN-α and IFN-γ on the persistently infected macrophage cell cultures. DRAW cells were treated with 250 ng/ml IFN-α or 25 ng/ml IFN-γ. A spectacular decrease of the infectivity was observed in IFN-treated DRAW cells, resulting in the complete elimination of the virus after 72 h with IFN-γ and after 96 h with IFN-α, whereas viral yields remained high (4.76 log_10_) in untreated DRAW cells after 96 h (Figure 2B). To ascertain that the IFN-treated DRAW cells were indeed virus-free, the cells were further cultivated for 30 days and regularly assayed for infectious virus. Because we never found any plaque, the persistent infection was indeed cleared in these cells, which we termed CDRAW.

### Viral infection upregulates RANTES in DRAW cells

**Figure 3:**
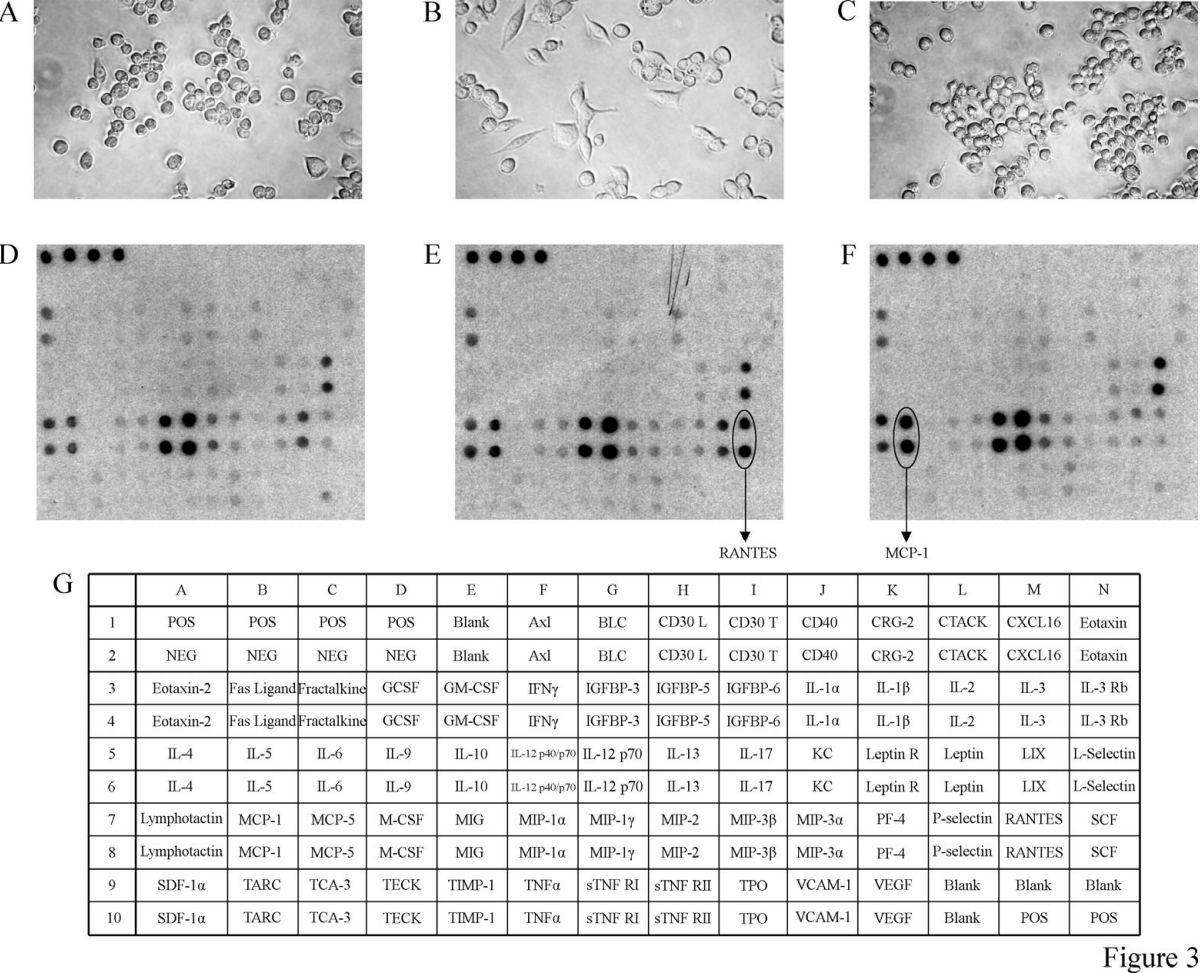
Phase-contrast images of (A) RAW; (B) DRAW and (C) CDRAW cells. Magnification: 400×. Protein array analysis of secreted cytokines by (D) RAW; (E) DRAW and (F) CDRAW cells. Cells were seeded at a density of 6 × 10^5 ^cells/well in 6-well plates. Culture supernatants were collected after 48 h and analyzed with RayBiotech's mouse cytokine antibody array III (G). Spots with differentially regulated cytokines are encircled.

In comparison with RAW cells, which displayed a round morphology (Figure 3A), we observed that DRAW cells showed a morphologic change in about 5 to 20% of the total cell population, resulting in an elongated phenotype (Figure 3B), which probably reflects an activation or differentiation process. In contrast, CDRAW cells that were cured from the persistent infection as a result of IFN-treatment, again acquired a round morphology (Figure 3C). No marked growth rate differences were observed between the different cell lines (results not shown).

To investigate whether the similar morphologic appearance between RAW and CDRAW cells would also be reflected by their cytokine expression pattern and whether this would be different from that of DRAW cells, we compared the cytokine profile of these cell lines. Culture supernatants of macrophages, seeded at 6 × 10^5 ^cells/well in 6-well plates, were collected after 48 h and analyzed for expression of cytokines and chemokines using a protein array. Among other cytokines, RAW macrophages constitutively secreted eotaxin-2, lipopolysaccharide-induced CXC chemokine (LIX), lymphotactin, monocyte chemoattractant protein-1 (MCP-1), macrophage inflammatory protein (MIP)-1α, MIP-1β, MIP-2 and P-selectin (Figure 3D). In addition to the cytokines and chemokines expressed by RAW macrophages, DRAW cells distinctly produced more of the chemokine RANTES (regulated upon activation, normal T-cell expressed and secreted) (Figure 3E). This difference in RANTES production between RAW and DRAW cells was also observed in culture supernatants collected at 96 h (results not shown). CDRAW cells, originally treated with IFN-α (Figure 3F), displayed a cytokine and chemokine expression pattern qualitatively and quantitatively comparable to that of RAW macrophages, including RANTES but with the exception of MCP-1 that was upregulated. The same results were obtained with CDRAW cells, originally treated with IFN-γ (results not shown).

## Discussion

Viral infections can result in the establishment of a persistent infection and this is quite often linked to a severe pathology, e.g., human immunodeficiency virus-related encephalopathy or hepatitis virus-induced liver injury [37,38].

Viral persistence has also been recognized to be a key determinant for the induction of TMEV-induced demyelination in mice that is studied as an experimental animal model for human MS [3,18]. However, knowledge about the mechanism by which TMEV persists is scanty as it is the result of a complex interaction between the virus and its host that is only partially understood [39]. In addition, the inability to abrogate a persistent TMEV infection in susceptible mice is a hindrance uncovering the exact role of viral persistence in the pathogenesis of demyelination and autoimmunity [19]. We previously described DRAW macrophages as an *in vitro *model to study TMEV persistence [20]. In this study, we assessed modulating the viral persistence in these macrophage cell cultures using several compounds that were selected for their established effect on picornavirus replication. Two different strategies, namely the upregulation as well as the downregulation of the virus infection were hereby considered.

In another study, we reported that 2-AP, an inhibitor of the double-stranded RNA-dependent protein kinase, enhances the replication of Theiler's GDVII strain but not that of the DA strain in RAW macrophages [21]. In line with these results, 2-AP didn't affect virus yields from DRAW cells. Neither did 2-FMC, an inhibitor of rhinovirus RNA synthesis [24]; hydantoin, an inhibitor of poliovirus post-synthetic protein cleavages and assembly [25]; levamisole, a compound known to potentiate the antiviral effect of interferon against encephalomyocarditis virus [26]; L-NAME and L-NMMA, two inhibitors of the cellular inducible NO synthase and resulting in increased coxsackie B virus titers [22,23], and pirodavir, a capsid-binding compound that inhibits rhinovirus uncoating [27].

Hemin is a metalloporphyrin that has been documented to increase poliovirus titers in persistently infected K562-Mu erythroleukemia cells resulting in a cytolytic infection [28]. Likewise, hemin-treated DRAW cells underwent lysis that was not observed in hemin-treated RAW macrophages. Hemin treatment of the former also led to an average fivefold increase of virus titers compared to untreated DRAW cells, which might be due to upregulation and/or spread of the infection as a result of the hemin-induced inhibition of interferon-mediated antiviral protection [31]. The latter effect may be related to the upregulation of ferritin, an iron-binding protein that can inhibit the transcription of IFN-α/β by suppression of the transcriptional activator nuclear factor-κB (NF-κB) [32,33]. Interestingly, Zoll *et al *[40] found that the L protein of mengovirus, like TMEV a member of the *Cardiovirus *genus, also suppresses the production of IFN-α/β through ferritin-mediated inhibition of NF-κB activation.

It has been speculated that the restricted replication of TMEV in macrophages might shield the virus from effective immune recognition and contributes to the establishment of the persistent infection [41-43]. Although hemin was found to increase viral replication, it also induced cell death, thereby potentially compromising its *in vivo *use because it could spread the infection beyond control and induce unwanted cell death.

The converse approach, consisting in the downregulation of the viral replication to eventually cure the persistently infected DRAW cells, may therefore be less hazardous. In that context, enviroxime, an inhibitor of polio- and rhinovirus RNA synthesis that presumably targets a replication complex component that interacts with the viral protein 3A(B) [34], and neutralizing mAb raised against the capsid protein VP1 of TMEV, were shown to exert an antiviral effect in DRAW cells. Added individually, both compounds reduced the virus titers approximately 10-fold. When combined together, a 100-fold decrease in virus yield was noticed, demonstrating additive antiviral activity that likely results from their different mode of action. Although the decrease of infectivity was maximally 2 log_10_, it must be said that we only added the compounds once (at the start of the experiment). It may be worthwhile to investigate the effect of multiple administrations, which might increase the antiviral efficacy.

We also explored the possibility of using IFNs to lower the infectious titers in DRAW cells. IFNs are produced by virus-infected cells and play a crucial role in the host's defense against viruses by conferring an antiviral state in neighboring, uninfected cells [36]. In this study, IFN-γ (type II IFN) and IFN-α (a type I IFN) were shown to inhibit viral replication to the point that no infectious virus was found anymore after 72 to 96 h, respectively. Others have shown the importance of IFNs in neuronal viral clearance and prevention of TMEV persistence using IFN- and IFN receptor-deficient mice [44,45]. However, this is the first report, as far as we know, demonstrating IFN-induced clearance of a persistent TMEV infection. These results indicate that there might be a therapeutic potential to cure mice persistently infected with TMEV. Apart from their antiviral effect, IFNs are also potent immunomodulators and Njenga *et al *[46] have shown that IFN-α/β treatment can result in the promotion of remyelination as well as in the aggravation of demyelination depending on the duration of the treatment.

Chemokines are chemotactic cytokines that are responsible for the migration and accumulation of leukocytes in specific tissue sites. Accumulating evidence indicates a role for chemokines in the pathogenesis of various CNS inflammatory diseases, including MS and virus-induced demyelination (for a review, see [47]). By comparison of the cytokine expression pattern between DRAW and uninfected RAW macrophages using protein arrays, we found one major difference, i.e., the upregulation of the chemokine RANTES in DRAW cells. Interestingly, other investigators have reported increased RANTES mRNA expression in the context of TMEV infections [48,49]. In CDRAW cells, on the other hand, where the persistent infection was cleared with IFN, RANTES showed the same low expression level as in uninfected RAW cells. This indicates that its upregulation in DRAW cells is related to the presence and/or replication of the virus. Interestingly, RANTES has also been detected in brain lesions of MS patients [50] and the RANTES gene might be linked with an increased genetic susceptibility to this disease [51]. In addition to its role as a chemoattractant, RANTES seems also important in viral clearance by mediating resistance against virus-induced death of macrophages [52] and its transcriptional upregulation is drastically antagonized by the leader protein of TMEV [53]. Therefore, the activation of RANTES might be a double-edged sword, contributing to antiviral defense at one hand and leading to inflammatory cell recruitment with immunopathologic injury on the other hand.

We also found an upregulation of the chemokine MCP-1 in CDRAW cells compared to RAW and DRAW macrophage cell cultures. Recently, Karpus *et al *[54] have shown the importance of this chemokine by inhibiting Theiler's virus induced-demyelination with anti-MCP-1 antibodies. Consequently, as with RANTES, MCP-1 seems to play a crucial role in the pathogenesis of demyelinating disease, but further research is necessary to unravel their exact contribution. Apart from the above mentioned cytokines, other cytokines might play a role in TMEV infection of macrophages as evidenced by recent work [55,56].

## Conclusion

We have identified several compounds that modulate viral replication in an *in vitro *model system for TMEV persistence by increasing or decreasing virus titers. Because there might be a rational basis for the upregulation as well as the downregulation of viral replication, these strategies now await further testing in an *in vivo *setting to address fundamental questions regarding persistent viral infection and immunopathogenesis. In addition, our results demonstrate the potential of DRAW cells to be used as a screening platform for the selection of known as well as future compounds for their effect on TMEV persistence.

## Materials and methods

### Cells

RAW264.7 cells, a mouse macrophage cell line derived from an Abelson murine leukemia virus-induced tumor in BALB/c mice, were kindly donated by T. Michiels (Christian de Duve Institute of Cellular Pathology, UCL, Belgium). DRAW macrophages were originally obtained by infection of RAW cells with 10 PFU/cell of Theiler's DA strain and ever since are persistently infected with this strain with the concomitant production of infectious virus [20]. CDRAW macrophages were obtained by treating DRAW cells with IFN-α or IFN-γ as a result of which the persistent infection was cleared. RAW, DRAW and CDRAW cells were grown in Dulbecco's modified Eagle medium (DMEM) with 2.5% fetal bovine serum (FBS).

L929 cells, originally derived from normal subcutaneous areolar and adipose tissue of a 100-day-old male C3H/An mouse and purchased from ATCC, were used for plaque assay. Cells were grown as monolayers in minimal essential medium supplemented with Earle's salts, nonessential amino acids, 1 mM sodium pyruvate, and 5% horse serum. All medium components were purchased from Invitrogen (Merelbeke, Belgium).

### Compounds

Compounds were dissolved as follows: stock solutions of 10 mg/ml of 2-FMC, enviroxime and pirodavir (kindly provided by Dr. Andries, Johnson and Johnson Pharmaceutical R&D, Beerse, Belgium), as well as hydantoin (Lilly Research Laboratories, Indianapolis, IN, USA) were made in dimethyl sulfoxide and diluted in medium, i.e., DMEM with 2.5% FBS, before use. Stock solutions of 10 mg/ml 2-AP; 4 mg/ml levamisole; 10 mg/ml L-NAME and 1 mg/ml L-NMMA were directly made in medium; hemin: a stock solution of 3.25 mg/ml was prepared by adding 1 ml of 1 M NaOH to 40 mg of hemin, followed by the addition of 10.1 ml DMEM and 1.2 ml of 1 M HCl. 2-AP, hemin, levamisole, L-NAME and L-NMMA were purchased from Sigma (Bornem, Belgium). Stock solutions were sterilized by filtration through 0.2 μm pore-size filters (Machery-Nagel, Düren, Germany). Stock solutions of 100 μg/ml recombinant murine IFN-α (HyCult biotechnology, Uden, The Netherlands) and 1 mg/ml recombinant murine IFN-γ (PeproTech, Rocky Hill, NJ, USA) were made in medium. Neutralizing mAb, originally obtained from Dr. Brahic (Institut Pasteur, Paris, France), was diluted in medium.

Experiments were performed in 96-well plates (Greiner Bio-One, Wemmel, Belgium) in a total volume of 200 μl consisting of 100 μl medium with cells and 100 μl medium containing the compound.

### Plaque assay

Infective titers were determined in culture supernatants and cells by a standard plaque assay on confluent L929 cells grown in 60 mm Petri dishes (Greiner Bio-One, Wemmel, Belgium) as described previously [20]. Samples, consisting of supernatants and cells, were analyzed after three rounds of freezing and thawing.

### Cell viability assay

Compound-induced cytotoxicity was evaluated in RAW and DRAW cells using the CellTiter-Blue cell viability assay (Promega, Leiden, The Netherlands) that measures the metabolic activity of cells based upon the reduction of the indicator dye resazurin into the highly fluorescent resorufin. Cells were seeded at 2.5 × 10^4 ^cells/well in black 96-well plates. The viability was determined at the start of the experiment and each following 24 h during 4 days according to the manufacturer's instructions. Briefly, after 2 h of incubation of the cells with the indicator dye at 37°C, the fluorescence was measured at an excitation wavelength of 530 ± 25 nm and an emission wavelength of 590 ± 35 nm with a Bio-Tek FL600 microplate fluorescence reader. Triplicate samples were assayed, background corrected and the results were expressed as a percentage of the values from untreated control cells.

### Cytokine protein arrays

Cytokine expression profiling was performed on culture supernatants from 6 × 10^5 ^cells using the mouse cytokine antibody array III from RayBiotech (Norcross, GA, USA) that allows the simultaneous detection of 62 different murine cytokines and chemokines (Figure 3G). Analysis was done according to the instructions of the manufacturer. Briefly, cytokine array membranes were first treated with blocking buffer, washed and then incubated with 1.5 ml of culture supernatants from either RAW, DRAW or CDRAW macrophages for 1.5 h. After washing, 1 ml of biotin-conjugated anti-cytokine detection antibodies was added. Following a further incubation of 1.5 h, the membranes were washed again and finally incubated with 2 ml of horseradish peroxidase-conjugated streptavidin for 2 h. The results were visualized on Kodak's Biomax MR X-ray film following enhanced chemiluminescence detection.

### Phase-contrast microscopy

Phase-contrast microscopy was performed with a Zeiss Axiovert100 microscope and photomicrographs were taken with an AxioCam MRc5 digital camera.

## Competing interests

The authors declare that they have no competing interests.

## Authors' contributions

SS participated in the experimental design, implementation and interpretation of results and performed all the experiments. SS is also the main contributing author of this manuscript. EM participated in cytokine protein arrays experiments and manuscript preparation. BR took part in discussing the results and supervised the study. RV helped with experimental design, data interpretation and supervised the study.

All authors read and approved the final manuscript.
